# Sensitive and Robust LC-MS/MS Assay to Quantify 25-Hydroxyvitamin D in Leftover Protein Extract from Dried Blood Spots

**DOI:** 10.3390/ijns7040082

**Published:** 2021-12-10

**Authors:** Sanne Grundvad Boelt, Lars Melgaard, Marta Jadwiga Thorbek, Nadia Sara Jensen MacSween, John J. McGrath, Arieh S. Cohen

**Affiliations:** 1 Center for Neonatal Screening, Department of Congenital Disorders—Clinical Mass Spectrometry Statens Serum Institut, Artillerivej 5, 2300 Copenhagen, Denmark; SGB@ssi.dk (S.G.B.); LMG@ssi.dk (L.M.); MAJB@ssi.dk (M.J.T.); NSJM@ssi.dk (N.S.J.M.); ACO@ssi.dk (A.S.C.); 2 National Centre for Register-Based Research, Department of Economics and Business Economics, Aarhus University, Fuglesangs Allé 26, Building 2640, DK-8210 Aarhus V, Denmark; 3 Queensland Centre for Mental Health Research, The Park Centre for Mental Health, Wacol, QLD 4076, Australia; 4 Queensland Brain Institute, University of Queensland, St. Lucia, QLD 4072, Australia

**Keywords:** LC-MS/MS, leftover protein extract, dried blood spot, vitamin D metabolites, 25OHD_3_, 25OHD_2_

## Abstract

Neonatal dried blood spots (DBS) provide a remarkable resource for biobanks. These microsamples can provide information related to the genetic correlates of disease and can be used to quantify a range of analytes, such as proteins and small molecules. However, after routine neonatal screening, the amount of DBS sample available is limited. To optimize the use of these samples, there is a need for sensitive assays which are integrated across different analytic platforms. For example, after DNA extraction, protein extracts are available for additional analyses. We describe a sensitive and robust LC-MS/MS method for 25-hydroxyvitamin D_2_ and 25-hydroxyvitamin D_3_ optimized for leftover protein extracts from DBS, which has excellent recovery, precision, and accuracy.

## 1. Introduction

Neonatal dried blood spots (DBS) have long been used for routine clinical screening for a range of congenital disorders in newborns [1]. If kept in biobanks, these samples provide a remarkable resource for risk factor epidemiology as these samples (a) are often part of a large set of unselected samples; (b) can provide DNA for genotyping [2]; (c) allow for the quantification of a wide range of analytes of interest (e.g., proteins and small molecules); and (d) can be linked with electronic health records and health registers [3]. However, a significant challenge using DBS is that only small volumes of clinical matrices are collected, and after routine neonatal screening, even less material is available for subsequent storage and research. Research groups are then faced with the challenge of using this precious resource efficiently. We had access to DBS protein extracts that had previously been used for genotyping [2]. We developed and validated a sensitive and robust LC-MS/MS approach capable of quantifying the two main vitamin D metabolites (25OHD_2_ and 25OHD_3_) in the leftover of the protein extract from DBS.

## 2. Methods

**Figure 1:**
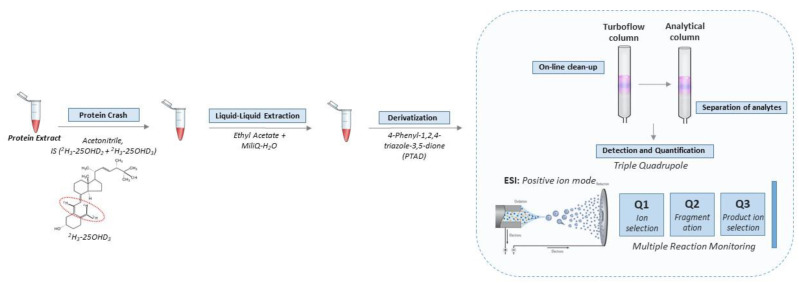
Workflow for quantification of vitamin D metabolites in leftover protein extract from DBS. Briefly, the vitamin D metabolites were extracted and derivatized with PTAD prior to entering the automated LC-MS/MS setup. The LC part consists of a combination of a TurboFlow column and an analytical column in order to reduce ion suppression and separate the vitamin D metabolites, respectively. Subsequently, detection and quantification were achieved in positive ion mode in a triple quadrupole mass spectrometer equipped with MRM. The approach was further optimized to be used in high throughput.

The standards, instrumentation, chromatographic condition (Table S1), tandem mass spectrometry detection (Figure S2) and quantification of the vitamin D metabolites were consistent with our previous methods for detecting vitamin D metabolites in DBS [4]. Full details of these steps are provided in the Supplement. The key steps in the workflow are shown in Figure 1. 

### 2.1. Protein Extract

The DBS containing either the stable isotope-labelled calibrator standard or quality controls or endogenous SRM^®^ 972 or internal controls were punched (2 mm × 3.2 mm punch per well equivalent to 6.56 µL whole blood) in Nunc^®^ MicroWell^TM^ 96 well polystyrene plates. Proteins were extracted by adding 130 µL of an in-house made extraction buffer (PBS containing 5 mL/L Tween20 and “Complete protease inhibitor cocktail with EDTA”—1 tablet dissolved per 25 mL of extraction buffer) to each well before the plates were shaken one hour at 450 rpm at room temperature.

### 2.2. Extraction of Vitamin D from Protein Extract

All protein extracts, including stable isotope-labelled calibrator standards, quality controls and endogenous SRM^®^ 972 and internal control DBS punches, were subjected to the same additional extraction procedure for vitamin D quantification. Samples were wrapped in aluminum foil to avoid contact with light. First, 30 µL of each sample was transferred to a Thermo Scientific 96-well NUNC microtiter plate before 120 µL internal standard (reconstituted in acetonitrile and diluted to a working solution of 1:100 compared to the kit insert) was added. The plate was placed on an orbital shaker (450 rpm) for 10 min at room temperature to facilitate protein precipitation. Next, the precipitated proteins were removed by centrifugation for 30 min at 4000× *g* rpm (4 °C). Subsequently, 80 µL of the supernatants were transferred into a 96 deep-well plate already containing 400 µL ethyl acetate and 180 µL deionized water for the liquid–liquid extraction procedure. All samples were mixed well using the “pipetting-mixing” function on an electronic pipette (20 cycles of pipetting 500 µL ups and down). Two phases were separated during centrifugation at 700× *g* rpm for 5 min (4 °C), before approximately 200 µL of the upper organic phase (containing the purified vitamin D metabolites) was transferred to a Thermo Scientific^TM^ WebSeal Plate+ 96-Well Glass-Coated Microplate. The samples were dried down in an Eppendorf Bench Top Concentrator Plus^TM^ (60 °C) before the vitamin D metabolites were derivatized with 20 µL of the commercial PTAD reagent (reconstituted in ethyl acetate and diluted to a working solution of 1:12). The plate was incubated on an orbital shaker (450 rpm) at room temperature for 30 min, after which the reaction was quenched by the addition of 50 µL ethanol. Samples were dried down in a concentrator before being reconstituted in 80 µL 1:1 acetonitrile/deionized water solution. The reconstitution was carried out by mixing the plate on an orbital shaker (450 rpm) for 10 min at room temperature. The plate was centrifuged at 3000× *g* rpm for 10 min at 4 °C and transferred to the autosampler. Subsequently, 40 µL was injected into the LC-MS/MS system. 

### 2.3. Validation of the Quantitative LC-MS/MS Method

The quantitative LC-MS/MS approach was validated in accordance with the Clinical and Laboratory Standards Institute’s approved guideline for liquid chromatography-mass spectrometry methods (C62-A). Linearity and calibration curve were evaluated using stable isotope-labelled calibration standards [5]. Precision and intermediate precision was analysed by performing intra- and inter-assay experiments using in-house endogenous internal controls. Intra- and inter-assay precision were determined by examining samples in triplicate within one assay and triplicates conducted in three consecutive days, respectively. To evaluate the accuracy, Standard Reference Material (Vitamin D Metabolites in Frozen Human Serum-SRM^®^ 972—from NIST) was converted to DBS in which the proteins were extracted and analysed according to the above approach. The performance of the assay was considered acceptable if the precision (%CV) at each concentration is <20% and the bias is ±20% of the expected reference value. Lower limit of detection (LOD) is determined when the peak height is three times the background noise. Lower limit of quantification (LOQ) was determined using dilutions of the lowest stable-isotope-labelled calibrator standard and with precision <20% and recovery within ±20%. 

The assay calibration was achieved by using a combination of the commercial external stable-isotope-labelled six-point calibration standards for both 25OHD_2_ and 25OHD_3_ (^2^H_6_-25OHD_2_ and ^2^H_6_-25OHD_3_) together with the different stable-isotope-labelled internal standards (^2^H_3_-25OHD_2_ and ^2^H_3_-25OHD_3_) [5]. See Supplementary Figure S1 for the chemical structure of the endogenous as well as stable-isotope-labelled versions of the two vitamin D metabolites. The stable-isotope-labelled internal standard was added during the extraction procedure in order to undergo processing identical to the stable-isotope-labelled calibration standards. To investigate the stability of the extracts, multiple freeze–thaw cycles and autosampler stability experiments were conducted using the stable-isotope labelled internal standard. To rule out any carry-over effect, blank samples after injection of the highest calibration standards were examined. 

## 3. Results

Based on the commercial calibration standards with known concentrations. regression equations for 25OHD_2_ and 25OHD_3_ were Y = 2.2338 × 10^−2^ [25OHD_2_] + 4.617 × 10^−3^; R^2^ = 0.9907 and Y = 2.492 × 10^−2^ [25OHD_3_] − 3.699 × 10^−3^; R^2^ = 0.9921, respectively. The analyte responses were linear within the calibration curves for both 25OHD_2_ and 25OHD_3_. In addition, the correlation between the known nominal concentrations (nmol/L) against the ratio of the back-calculated concentrations (nmol/L) relative to the internal standard which are based on the MRM responses, were plotted too. The regression equations were Y = 0.9991 × [25OHD_2_] + 0.8879; R^2^ = 0.9988 and Y = 1.007 × [25OHD_3_] + 0.1279; R^2^ = 0.9990 25OHD_2_ and 25OHD_3_, respectively. The relative error (%), between the nominal and back-calculated concentration, fluctuated within ±6%, which is well within the CLSI limit of ±15%. The precision was investigated by quantifying the concentration of endogenous 25OHD_2_, 25OHD_3_ and total (25OHD_2_ + 25OHD_3_) in full blood concentrations in six in-house internal controls. Supplementary Table S2 shows intra- and inter-assay precision recoveries and %CV. In summary, the %CV for 25OHD_3_ was in the range of 0.5–7.7% and the %CV for the total full-blood concentrations ranged from 1.1–8.4%, while %CV for 25OHD_2_ ranged between 3.9–17.1%. The suboptimal %CV for 25OHD_2_ mainly reflects the low concentration of endogenous 25OHD_2_ in the samples, which has been observed in previous DBS-related studies [4,6]. 

Intermediate precision was obtained by quantifying the concentration of three stable-isotope-labelled external commercial quality controls with a low, medium and high concentration of each vitamin D metabolite (^2^H_6_-25OHD_2_ and ^2^H_6_-25OHD_3_). All three quality controls for each of the two metabolites were quantified twenty-three times, measured over a period of approximately four months. Geometric means, SD, %CV, relative error and accuracy were calculated for all measurements and are summarized in Supplementary Table S3. SD for the six quality controls is in the range of 0.8–4.9 and %CV in the range of 4.7–7.2%, demonstrate that the approach is suitably stable. Additionally, the relative error fluctuates in the range from 1.3–6.6% and an accuracy range from 101.3–107%, which demonstrates a stable intermediate precision of the assay over an extended period of time.

To investigate the accuracy of the new method, SRM^®^ 972 serum from NIST were mixed 1:1 with purified erythrocytes and spotted onto filter paper before the proteins were extracted followed by the extraction, derivatization and quantification of the endogenous levels of 25OHD_2_ and 25OHD_3_ as described above. The evaluation of the SRM^®^ 972 from NIST demonstrated excellent accuracy of the approach (92–105%) (Supplementary Table S4). Additionally, the acceptable %CV that ranges between 4.7–13.2% and relative errors fluctuating in the range of −7.9–5.7% indicates that the method was stable and accurate.

To estimate the LOQ for our new assay, dilutions of the lowest stable-isotope-labelled calibrator standards for both vitamin D metabolites (^2^H_6_-25OHD_2_ and ^2^H_6_-25OHD_3_) were prepared and quantified. The concentrations of each vitamin D metabolite (nmol/L) were plotted against the relative errors (Supplementary Figure S3). The results demonstrated that the approach was able to detect a concentration of both 25OHD_2_ and 25OHD_3_ down to approximately 5 nmol/L in the protein extracts (values were adjusted to reflect estimated full blood concentration). 

The stability of the extracts was investigated using the six in-house internal controls. In the first stability study, the protein extracts were exposed to six freeze/thaw cycles before the endogenous vitamin D metabolites were extracted, derivatized, separated and quantified. In the second stability study, the samples containing the extracted and derivatized samples were kept at 5 °C up to 24 h in the autosampler before they were quantified. Each experiment was conducted in triplicate. The geometric means of the concentrations and the %CV and %bias, compared to fresh protein extract, were calculated and summarized in Supplementary Table S5. In summary, the freeze–thaw cycles resulted in lower concentrations and the autosampler storage resulted in increased concentrations. However, the effects associated with these biases were associated with acceptable CV% (below 15%). Furthermore, no carry-over was observed for either 25OHD_2_ and 25OHD_3_ based on the criterion that the signal for a blank sample injected directly after the highest calibrator standard was less than 20% of the signal of the lowest calibrator standard. 

## 4. Discussion

We report a sensitive and robust approach for LC-MS/MS quantification of small molecule (vitamin D metabolites) based on leftover protein extract from neonatal DBS. We demonstrated that the method has acceptable precision and accuracy and is sensitive and robust. As the assay was optimized to use only 30 uL of the leftover protein extract from DBS (equivalent to 1.51 uL of whole blood), it is possible to combine this new assay with other analytical assays, such as genotyping and protein quantification. With only minor adjustments, this approach could also be used for other small molecules of interest in an efficient manner. 

When interpreting results from this new assay, there are several important limitations to take into account. Neonatal hematocrit is known to influence assays based on DBS [7], and information on the hematocrit for DBS is not routinely available. In order to estimate serum values of the endogenous levels of vitamin D, full blood concentrations are multiplied by a factor of 2.56 based on the assumption of a capillary hematocrit of 60% [8]. BSA has been used in protein extracts from DBS in order to stabilize proteins and thereby improve the sensitivity of protein-based analytes. However, BSA may contain (exogenous) bovine 25OHD_2_ and 25OHD_3_ [9]. Therefore, we recommend that laboratories should avoid methods that involve BSA if vitamin D is of interest. 

Although several other sensitive LC-MS/MS vitamin D quantification assays for DBS already exist, none have reported quantification of vitamin D metabolites in protein extracts from DBS. The quantification of the 25OHD_2_ and 25OHD_3_ from protein extracts facilitates the assessment of a panel of vitamin D-related measures (e.g., the quantification of the concentration of vitamin D binding protein, genetic variants related to vitamin D metabolism). We believe that our new assay will facilitate this type of integrated research in neonatal archived DBS.

## Data Availability

The data presented in this study are available on request from the corresponding author.

## Supplementary Materials

The following are available online at https://www.mdpi.com/article/10.3390/ijns7040082/s1.
