# Supplementary material for: Sensitive and Robust LC-MS/MS Assay to Quantify 25-Hydroxyvitamin D in Leftover Protein Extract from Dried Blood Spots
Source: Int J Neonatal Screen. 2021 Dec 10;7(4):82. doi: 10.3390/ijns7040082 (PMC8704356; doi:10.3390/ijns7040082)
Supplement: Supplementary file 1 [file IJNS-07-00082-s001.zip › IJNS-1476396-supplementary.pdf]

Sensitive and robust LC-MS/MS assay to quantify 25-hydroxyvitamin D in leftover protein extract from dried blood spots

SUPPLEMENTARY MATERIAL

Contents

Supplementary methods 2

Supplementary Table 1........ 6

Supplementary Table 2........ 7

Supplementary Table 3........ 8

Supplementary Table 4........ 9

Supplementary Table 5........ 10

Supplementary Figure 1 11

Supplementary Figure 2 12

Supplementary Figure 3 13

References........ 13

Supplementary methods

Standards

MS/MS Vitamin D kit (containing lyophilized serum of six point stable-isotope labelled calibrator standards ($^2H_6$-25OHD$_2$ and $^2H_6$-25OHD$_3$), three stable-isotope labelled quality controls ($^2H_6$-25OHD$_2$ and $^2H_6$-25OHD$_3$) and stable-isotope labelled Internal Standard ($^2H_3$-25OHD$_2$ and $^2H_3$-25OHD$_3$)), *MS/MS Vitamin D Derivatization Box* (containing PTAD:4-Phenyl-1,2,4-triazoleline -3,5-dione derivatization reagent, quench solution and HPLC solvent additive (methyl amine)) and *MS/MS Vitamin D Maintenance Set* (containing endogenous 25OHD$_2$ and 25OHD$_3$) were purchased from PerkinElmer (Turku, Finland). The chemical structure of both the endogenous 25OHD$_2$ and 25OHD$_3$ and the stable-isotope labeled calibrator standards and quality controls of 25OHD$_3$ are shown in Figure S2. Standard reference material (SRM ®972) for both 25OHD$_2$ and 25OHD$_3$ were purchased from National Institute of Standards and Technology (NIST) (Gaithersburg, United States). Methanol (99.9% LC-MS Grade) and 2-Propanol (Optima®LC-MS) were purchased from Thermo Scientific (Slangerup, Denmark). Acetonitrile was purchased from Sigma-Aldrich (Brøndby, Denmark). Formic Acid was purchased from VWR & Bie & Berntsen A/S (Herlev, Denmark). Water was purified on Millipore milli-Q system.

Instrumentation

The LC system is a Thermo TLX2 Turboflow system, comprised of a CTC Analytics HTS PAL autosampler, a dual LC system (one Agilent 1200 quartinary and one Agilent 1200 binary pump) and two Thermo Scientific hot pocket column heaters. The LC systems are interfaced with a triple quadrupole mass spectrometer (Thermo Scientific TSQ Quantiva) equipped with a heated electrospray ionization probe. The LC system is controlled by Aria MX Direct Control software, whereas the mass spectrometer is controlled in the TSQ Quantiva Tune Application software (version 2.0.1292.15). Thermo TraceFinder™ 3.2 application software is used to acquire and process data.

Chromatographic condition

To minimize sample preparation time and reduce ion suppression, an automated online chromatographic cleaning was accomplished using a Thermo Scientific TurboFlow Technology Cyclone P column (0.50 x 50mm). The patented TurboFlow column efficiently removes large molecules, which are the primary interference in a biological matrix, by performing a combination of the principle behind size exclusion and turbulent flow chromatography. The optimized turbulence and diffusion of large and small molecules in the column, combined with the specific chemistry inside the pores in the particles developed to retain the small molecules of interest, enables a fast and efficient separation of the molecules. Here, the large molecules quickly flush to waste, while the small molecules of interest elute to the second analytical column when the mobile phase changes. The second column is a Hypersil Gold Reverse-Phase (RP) analytical column (50 x

2.1mm, 3μM particle size, Thermo Scientific), which is used to separate analytes according to the hydrophobicity. The analytical column was kept at 40°C in a hot pocket column heater. The injection volume was 40μL. The mobile phases through the two columns were gradients of Solvent A (25% methanol and 250μL/L additive), solvent B (100% methanol and 250μL/L additive) and solvent C (25% methanol, 25% acetonitrile, 25% isopropanol, 25% milliQ-H_2O and 2mL/L Formic Acid) and comprise an analytical and column cleaning gradient, respectively. The total method duration for the chromatographic separation is 6.5min. The chromatographic conditions and the step gradient used are shown in Table S1. Both LC-systems were used in parallel with staggered injections in order to maximize run-time efficiency.

Tandem mass spectrometry detection

Detection of the endogenous vitamin D metabolites $25OHD_2$ and $25OHD_3$, the stable-isotope labeled calibrator standards and the quality controls ($^2H_6$-$25OHD_2$ and $^2H_6$-$25OHD_3$) and their respective stable-isotope labeled internal standards ($^2H_3$-$25OHD_2$ and $^2H_3$-$25OHD_3$) was achieved by MS/MS detection in positive ion mode as methyl amine adducts. The MRM transitions for precursor/product ion used were m/z = 607.3 → 298.1 for 25-hydroxy D_3, m/z = 619.4 → 298.1 for 25-hydroxy D_2, m/z = 613.3 → 298.1 for $^2H_6$-25-hydroxy D_3, m/z = 625.3 → 298.1 for $^2H_6$-25-hydroxy D_2, m/z = 610.3 → 301.1 for $^2H_3$-25-hydroxy D_3 and m/z = 622.3 → 301.1 for $^2H_3$-25-hydroxy D_2. The optimal MS operating conditions were as follows: the static spray voltage = 3.5kV, the ion transfer tube and vaporizer temperature were set to 350°C. The sheath and auxiliary gas were set at 50 and 15 arbitrary units, nitrogen was used for both the sheath and auxiliary gases. Argon was used as collision gas at a pressure of 1.5m Torr. For $25OHD_3$, $^2H_6$-$25OHD_3$ and $^2H_3$-$25OHD_3$ the collision energy was 19 V. For $25OHD_2$, $^2H_6$-$25OHD_2$ and $^2H_3$-$25OHD_2$ the collision energy was 17 V.

Quantification of the vitamin D metabolites

Both the endogenous and stable-isotope labelled versions of the two main vitamin D metabolites ($25OHD_2$ and $25OHD_3$) were determined in Thermo TraceFinder^{TM} 3.2 application software using the metabolites' respective MRM transitions for their precursor and product ions described above. The chemical derivatization reaction between the vitamin D metabolites and PTAD reagent generates a new chiral carbon atom, resulted in the formation of two characteristic diasteromers – the major 6S-isomer and the minor 6R-isomer. While both diasteromers can be separated in this commercial vitamin D kit [1], peak integration of both signals combined was performed for quantitative determination of the vitamin D metabolites. A representative MRM [illegible] derivatized with PTAD showing the combined signals for the minor 6R-isomer and major 6S-isomer is shown in Figure 3. As described, a stable-isotope labelled internal standard ($^2H_3$-$25OHD_2$ and $^2H_3$-$25OHD_3$) is added to all samples, including both the stable-isotope labelled calibrator standards and external controls ($^2H_6$-$25OHD_2$ and $^2H_6$-$25OHD_3$) as well as samples with unknown levels of endogenous vitamin D. For quantification of the unknown concentration of endogenous vitamin D metabolites ($25OHD_2$ and $25OHD_3$), the ratio of the analytes to the internal standard signal is compared to the calibration

curve. The MS/MS Vitamin D kit utilizes isotope labelled calibrators which are more heavily labelled than the internal standards. The advantage to this approach is that the calibrators can be based on native serum with endogenous levels of vitamin D as the native levels will not be detected in the transitions used to measure the calibrators

Tandem mass spectrometry detection

Detection of the endogenous vitamin D metabolites $25OHD_2$ and $25OHD_3$, the stable-isotope labeled calibrator standards and the quality controls ($^2H_6$-$25OHD_2$ and $^2H_6$-$25OHD_3$) and their respective stable-isotope labeled internal standards ($^2H_3$-$25OHD_2$ and $^2H_3$-$25OHD_3$) was achieved by MS/MS detection in positive ion mode as methyl amine adducts. The MRM transitions for precursor/product ion used were m/z = 607.3 → 298.1 for 25-hydroxy D_3, m/z = 619.4 → 298.1 for 25-hydroxy D_2, m/z = 613.3 → 298.1 for $^2H_6$-25-hydroxy D_3, m/z = 625.3 → 298.1 for $^2H_6$-25-hydroxy D_2, m/z = 610.3 → 301.1 for $^2H_3$-25-hydroxy D_3 and m/z = 622.3 → 301.1 for $^2H_3$-25-hydroxy D_2. The optimal MS operating conditions were as follows: the static spray voltage = 3.5kV, the ion transfer tube and vaporizer temperature were set to 350°C. The sheath and auxiliary gas were set at 50 and 15 arbitrary units, nitrogen was used for both the sheath and auxiliary gases. Argon was used as collision gas at a pressure of 1.5m Torr. For $25OHD_3$, $^2H_6$-$25OHD_3$ and $^2H_3$-$25OHD_3$ the collision energy was 19 V. For $25OHD_2$, $^2H_6$-$25OHD_2$ and $^2H_3$-$25OHD_2$ the collision energy was 17 V.

Quantification of the vitamin D metabolites

Both the endogenous and stable-isotope labelled versions of the two main vitamin D metabolites ($25OHD_2$ and $25OHD_3$) were determined in Thermo TraceFinder™ 3.2 application software using the metabolites' respective MRM transitions for their precursor and product ions described above. The chemical derivatization reaction between the vitamin D metabolites and PTAD reagent generates a new chiral carbon atom, resulted in the formation of two characteristic diasteromers – the major 6S-isomer and the minor 6R-isomer. While both diasteromers can be separated in this commercial vitamin D kit [1], peak integration of both signals combined was performed for quantitative determination of the vitamin D metabolites. A representative MRM chromatogram of endogenous $25OHD_3$ derivatized with PTAD showing the combined signals for the minor 6R-isomer and major 6S-isomer is shown in Supplementary Figure 2. As described, a stable-isotope labelled internal standard ($^2H_3$-$25OHD_2$ and $^2H_3$-$25OHD_3$) is added to all samples, including both the stable-isotope labelled calibrator standards and external controls ($^2H_6$-$25OHD_2$ and $^2H_6$-$25OHD_3$) as well as samples with unknown levels of endogenous vitamin D. For quantification of the unknown concentration of endogenous vitamin D metabolites ($25OHD_2$ and $25OHD_3$), the ratio of the analytes to the internal standard signal is compared to the calibration curve. The MS/MS Vitamin D kit utilizes isotope labelled calibrators which are more heavily labelled than the internal standards. The advantage to this approach is that the calibrators can be based on native serum with endogenous levels of vitamin D as the native levels will not be detected in the transitions used to measure the calibrators. With respect to method validation, linearity was evaluated using

the commercial calibration standards with known concentrations; six stable-isotope labelled vitamin D metabolites ($^2H_6$-25OHD$_2$ and $^2H_6$-25OHD$_3$) in the range from approximately 5-180nmol/L (full blood concentrations). To demonstrate the linearity for the assay, the concentration as a function of analyte relative to internal standard responses were plotted.

There are several ways to determine Limit of detection (LOD) and Limit of quantification (LOQ). Here, LOD was determined when the peak height was 3 times the background noise, whereas LOQ was based on the precision and recovery requirements. Although the LOD value in fact is much lower, the LOQ is the primary interest for the new method. Therefore, LOQ was determined where it meets an acceptable precision (relative error <15%), according to the Clinical and Laboratory Standards Institute´s approved guideline for liquid chromatography-mass spectrometry methods (C62-A).

Statistical calculations

All experiments presented here are conducted at least in triplicate. Geometric means (mean), standard deviation (±SD), coefficient of variation (%CV), Relative Error (and %bias) are calculated using the following equations:

(1) $\bar{X}_{\text{geom}} = \sqrt[n]{\prod_{i=1}^{n} x_i} = \sqrt[n]{x_1 x_2 \ldots x_n}$

(2) $\pm\text{SD} = \sqrt{\frac{\sum(x_i - \bar{x})^2}{n-1}}$

(3) $\%\text{CV} = = \frac{\pm SD}{\bar{x}_{geom}} \cdot 100\%$

(4) $\%\text{Relative Error} = \frac{Back\text{-}calculated\ concentration - nominal\ concentration}{nominal\ concentration} \cdot 100\%$

wherein $\bar{x}$ = the average of all values, x_i = each value, n = the number of values.

DBS Calibration standards, quality control samples, SRM® 972 samples and internal standards

The stable-isotope labelled calibration standards and quality controls for 25OHD$_3$ and 25OHD$_2$ were prepared as follows. Human blood was collected in 6.0 mL EDTA coated BD vacutainer tubes and was gently inverted and kept cold on ice. The erythrocytes were isolated by aspirating plasma after centrifuging the blood samples at 805 rcf for 15min. at 4°C. Subsequently, the erythrocyte pellet was washed three times with a 0.9 mg/L NaCl MilliQ-H_2O solution before the erythrocytes were hemolyzed by sonication for 20 min. at 15°C. Subsequently, the commercial lyophilized serum containing stable-isotope labelled six-point calibrator standards and the three stable-isotope labelled quality controls were reconstituted in deionized water and mixed at 600 rpm on an orbital shaker for 1 hour. The reconstituted stable-isotope labelled calibrator standards and quality controls and the endogenous SRM® 972 samples were mixed 1:1 with the hemolyzed erythrocytes, before they were spotted onto filter paper in aliquot of 75 µL. In addition, six freshly taken blood samples from individuals (males and females from a range of ages), were taken and spotted onto filter paper in aliquots of 75 µL as internal controls. The blood on the filter paper was allowed to dry overnight at room temperature.

Supplementary Table 1

TurboFlow Column					*RP-Analytical Column*		
Time (min)	*% A*	*%B*	*%C*	*Flow (mL/min)*	*% A*	*%B*	*Flow (mL/min)*
0.00	*100*	-	-	*1.50*	*100*	-	*0.50*
0.50	-	*100*	-	*0.15*	*100*	-	*0.45*
2.00	-	*100*	-	*1.00*	*25*	*75*	*0.40*
4.00	-	-	*100*	*1.00*	*25*	*75*	*0.40*
5.50	-	-	*100*	*1.00*	-	*100*	*0.50*
6.00	*100*	-	-	*1.00*	-	*100*	*0.50*
6.50	*100*	-	-	*1.00*	*100*	-	*0.50*

Table S1: Chromatographic conditions for the online clean-up and separation of the analytes on the TurboFlow column and the RP analytical column in the presented approach. The step gradients of the mobile phases through the two columns comprise an analytical and column cleaning gradient. These gradients are illustrated by showing the flow rate (mL/min) and the percent content of each solvent (A-C) used at a given time point during the run that has a total duration of 6.5 minutes. Solvent A (25% methanol and 250µL/L additive, Solvent B (100% methanol and 250µL/L additive) and Solvent C (25% methanol, 25% acetonitrile, 25% isopropanol, 25% milliQ-H_2O and 2mL/L Formic Acid).

Supplementary Table 2

	Intra-assay precision			*Inter-assay precision*		
	Mean	± SD	%CV	Mean	± SD	%CV
**25-hydroxy D_3**						
Internal control 1	31.2	± 1.2	3.9	31.1	± 2.3	7.3
Internal control 2	34.3	± 0.5	1.5	34.3	± 0.2	0.5
Internal control 3	36.6	± 0.8	2.2	36.6	± 1.7	4.7
Internal control 4	45.3	± 3.5	7.7	45.3	± 2.3	5.0
Internal control 5	49.2	± 1.3	2.5	49.2	± 0.6	1.2
Internal control 6	83.5	± 6.9	8.3	83.5	± 4.1	4.9
**25-hydroxy D_2**						
Internal control 1	0.79	± 0.10	12.7	0.79	± 0.10	12.7
Internal control 2	0.90	± 0.03	3.9	0.90	± 0.03	3.9
Internal control 3	1.58	± 0.10	6.5	1.57	± 0.10	6.5
Internal control 4	0.72	± 0.05	7.3	0.72	± 0.05	7.3
Internal control 5	1.00	± 0.08	8.9	1.00	± 0.08	8.9
Internal control 6	0.94	± 0.16	17.2	0.94	± 0.16	17.1
**Total full blood**						
Internal control 1	32.0	± 1.3	4.2	31.9	± 0.2	0.1
Internal control 2	35.2	± 0.5	1.4	34.6	± 0.4	0.3
Internal control 3	38.1	± 0.9	2.2	37.1	± 0.3	0.4
Internal control 4	46.1	± 3.6	7.7	45.6	± 0.1	0.5
Internal control 5	50.2	± 1.3	2.7	49.5	± 0.9	0.2
Internal control 6	84.4	± 7.1	8.4	83.8	± 0.1	0.1

Table S2: Summary of intra-assays and inter-assays precision for $25OHD_3$, $25OHD_2$ and the total 25OHD ($25OHD_3$ + $25OHD_2$) based on full blood levels in six in-house internal control samples. Inter-assays and intra-assays were both performed in triplicates. The geometrical mean (mean) of the concentration (nmol/L), standard deviation (±SD) and coefficient of variations (%CV) are calculated and shown in the table. The concentrations are full blood values.

Supplementary Table 3

Analytes	*PerkinElmer specification*		*LC-MS/MS*			*Relative Error*	*%Accuracy*
	Value (nmol/L)	±SD	Mean (nmol/L)	±SD	%CV		
**25-hydroxy D_3**							
Quality Control C1	10.9	2.0	11.3	0.8	7.2	3.9	104.0
Quality Control C2	44.2	7.9	47.2	2.2	4.7	6.6	107.0
Quality Control C3	89.5	16	92.9	4.9	5.3	3.6	103.7
**25-hydroxy D_2**							
Quality Control C1	11.1	1.8	11.6	0.8	7.2	4.3	104.5
Quality Control C2	44.8	7.2	46.4	2.8	6.1	3.6	103.7
Quality Control C3	91.0	15	92.2	4.8	5.2	1.3	101.3

Table S3: Summary of intermediate precision results. Three different commercial external quality controls of $25OHD_2$ and $25OHD_3$ with known low, medium and high concentrations were quantified twenty-three times over a period of approximately four months in daily use. Geometrical means (mean) of the quantified concentrations (nmol/L), standard deviation (±SD) and coefficient of variations (%CV) are calculated and compared to the commercial certified values whereafter the relative error and accuracy were calculated. The concentrations are full blood values.

Supplementary Table 4

Analytes	*NIST Specification*		*LC-MS/MS*			*Relative Error*	*%Accuracy*
	Certified value (nmol/L)	±SD	Mean (nmol/L)	±SD	%CV		
**25-hydroxy D_3**							
SRM®972 Level 1	59.6	2.1	60.5	1.4	4.7	1.6	101.6
SRM®972 Level 2	30.8	1.5	32.5	1.8	11.2	5.7	105.6
SRM®972 Level 3	46.2	2.8	46.8	3.1	13.2	1.3	101.3
SRM®972 Level 4	176.4	4.9	172.2	8.6	10.0	-2.4	97.6
**25-hydroxy D_2**							
SRM®972 Level 1	-	-	-	-	-	-	-
SRM®972 Level 2	4.1	0.19	4.1	0.1	6.2	0.1	100.1
SRM®972 Level 3	64.1	4.8	59.8	2.9	9.9	-6.6	93.4
SRM®972 Level 4	5.81	0.52	5.3	0.2	8.3	-7.9	92.0

Table S4: Summary of the accuracy results. Geometrical mean (mean) of the concentration (nmol/L), standard deviation (±SD) and coefficient of variations (%CV) are calculated and compared to commercially certified values after which the relative error and accuracy estimates were calculated. The certified values for the NIST SRM ®972 samples are serum values. Therefore, the quantified full blood levels with the presented approach were corrected to serum values and shown in the table.

Supplementary Table 5

	Fresh (nmol/L)	*%CV*	*Freeze/thaw (nmol/L)*	*%CV*	*%Bias*	*Autosampler (nmol/L)*	*%CV*	*%Bias*
C1	36.6	7.5	33.9	3.1	-7.3	38.9	5.1	6.6
C2	83.5	12.2	75.1	4.9	-10.3	89.4	3.7	7.0
C3	34.3	4.7	33.5	8.8	-2.4	39.9	6.3	16.5
C4	49.2	4.9	46.8	9.5	-4.9	51.7	0.7	5.2
C5	45.3	9.8	43.4	5.4	-4.3	50.4	2.8	11.2
C6	31.2	8.7	27.5	8.6	-11.9	32.3	11.3	3.6

Table S5: Summary of stability data. Two different stability studies were conducted. In the first, the protein extracts from six internal control samples were exposed to 6 freeze/thaw cycles before $25OHD_2$ and $25OHD_3$ were extracted, derivatized and quantified. In the second, the extracted and derivatized $25OHD_2$ and $25OHD_3$ were kept at 5°C for 24 hours in the autosampler before they were analyzed and quantified. Each stability study was conducted in triplicate. Geometrical means (mean) of the quantified concentrations (nmol/L) are calculated and compared to concentration quantified from fresh protein extracts whereafter the %CV and %bias were calculated and shown in the table. The concentrations are full blood values.

Supplementary Figure 1

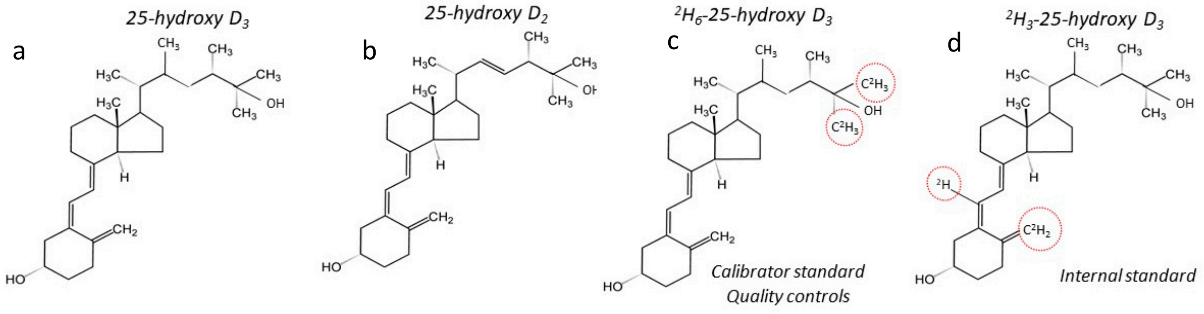

Figure S1: Chemical structure of the two vitamin D metabolites (25OHD$_2$ and 25OHD$_3$) measured in the assay. A-B) Illustrate the endogenous versions of both 25OHD$_2$ and 25OHD$_3$. C-D) Demonstrate the positions in 25OHD$_3$ of the incorporated stable-isotope labelled deuterium atom in the external commercial calibrator standard/quality controls and the internal standard, respectively. The positions of the incorporations are the same in the 25OHD$_2$ vitamin D metabolite. The stable-isotope labelled calibrator standard/quality controls are used to reduce the level of interfering compounds, whereas the stable-isotope labelled internal standards are added to normalize for sample preparation and instrumental variability, both improving the sensitivity of the approach. Although the retention times for the stable-isotope labelled metabolites are identical to their corresponding endogenous ones, there is a clear distinction between them due to the mass differences shown here.

Supplementary Figure 2

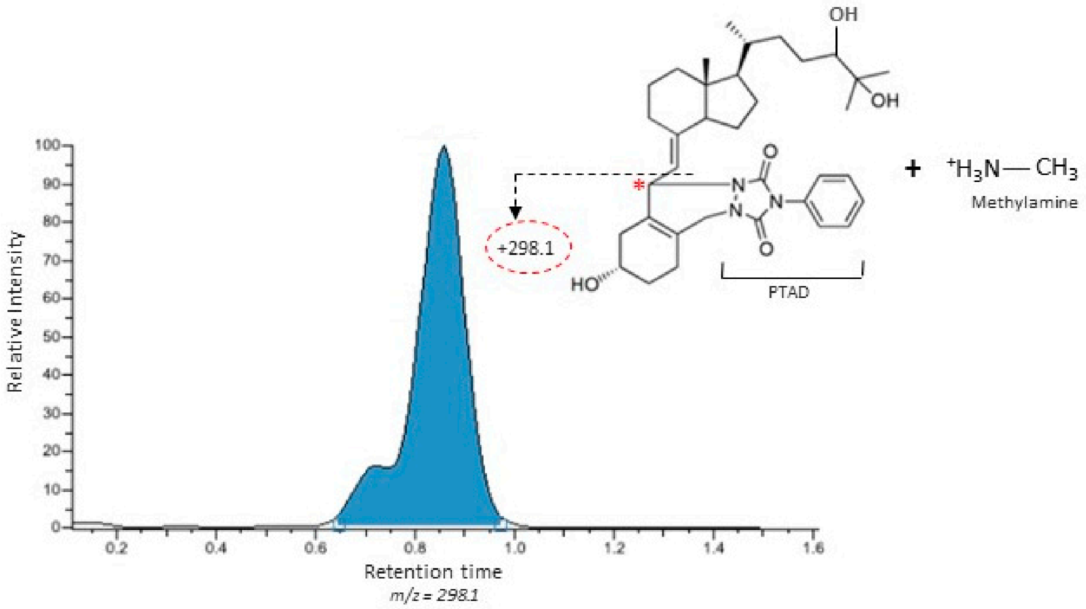

Figure S2: A representative LC-MS/MS chromatogram of the product ion of an endogenous 25OHD$_3$ vitamin D metabolite derivatized with PTAD acquired in MRM mode. The PTAD derivatization of the metabolites and the presence of methyl amine in the solvent is used to improve the ionization effect and MS/MS signal intensity. The chemical modification of the metabolites with PTAD generates a chiral carbon atom (marked *) and subsequently the formation of two diasteomers; the minor 6R-isomers and the major 6S-isomer.

Supplementary Figure 3

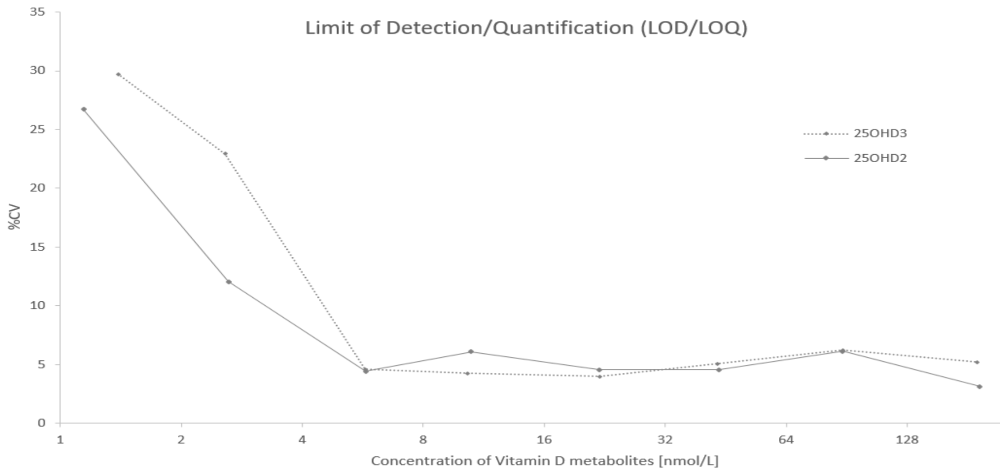

Figure S3: Determination of the Limit of Detection/Quantification(LOD/LOQ). To determine LOD/LOQ for the new method, the external commercial calibrator standards with known concentration (nmol/L) of $25OHD_2$ and $25OHD_3$ were used. In addition to the six calibrator standards with known concentrations, dilutions of the lowest calibrator standards were prepared. The concentrations (nmol/L) of the commercial calibrator standard were plotted against the %CV in order to find limit of detection. The concentrations are full blood values.

References

1. PerkinElmer. Rapid Quantitative Analysis of Vitamin D Metabolism with Nanogram Detection (25-Hydroxy D2 and 25-Hydroxy D3 in Human Serum. Available online: https://resources.perkinelmer.com/lab-solutions/resources/docs/app_msms_rapidquantitativeanalysisofvitamindmetabolism.pdf (accessed on 1/11/2020).
